# Supplementary material for: Assessment of healthcare financing by older adults during routine consultations in Cameroon
Source: PLOS Glob Public Health. 2026 Apr 24;6(4):e0006197. doi: 10.1371/journal.pgph.0006197 (PMC13108730; doi:10.1371/journal.pgph.0006197)
Supplement: Attachment — Submitted filename: New Microsoft Word Document (2).docx [file pgph.0006197.s003.docx]

**PEER REVIEW REPORT**

This study examines healthcare financing challenges faced by older adults in Cameroon, where universal health coverage is absent. Conducted between March and April 2023 across four health facilities in Yaoundé, the research involved 170 participants aged 60 and above, with a mean age of 68.4 years. The study population was predominantly female and lived in socially and economically vulnerable conditions, with women particularly affected.

The findings reveal that cardiovascular, musculoskeletal, and urological conditions accounted for over 70% of consultation reasons, while hypertension, rheumatic disorders, and diabetes were the most common comorbidities. The average cost of a routine medical consultation, including medications and diagnostic tests, was 42,958 FCFA (approximately €65.49), representing a substantial financial burden that sometimes exceeded half the national minimum monthly wage. Transportation, medications, and diagnostic tests constituted the major expense components.

The study demonstrates that healthcare financing relies almost entirely on out-of-pocket payments, with 43.5% of cases financed through direct household payments and 47.1% through family support, primarily from children. Only 8.2% of participants had health insurance coverage, with notable disparities based on marital status and employment status. Despite family solidarity mechanisms, over half of the older adults reported difficulty covering all medical expenses, leading 38.5% to resort to self-medication and 15.4% to use traditional medicine.

The research highlights the critical need for structural reforms to address these challenges. The authors recommend implementing a subsidized geriatric care package, developing insurance products tailored to the informal sector, deploying mobile geriatric clinics, and strengthening therapeutic education programs. These measures are essential to ensure equitable healthcare access for older adults in Cameroon, particularly given the rapid demographic transition and the limitations of the current social protection system, which covers only about 10% of the population and excludes the vast majority of informal sector workers.

**Remarks**

Great work overall.

1) Introduction- address the need for the study. Any similar study in the past? Why was Yaounde chosen?
2) Table 1- Format properly. Remove the commas and insert decimal points for percentages.
3) Table 2 – Also needs the same correction.
4) Table 1- Age column totals up to 99.9%.
5) Figure 1- Total percentage adds up to 131.3%.
6) Figure 2- Total percentage adds up to 109.8%.
7) Discussion- Please mention the strengths and limitations in a paragraph exclusively.
8) Conclusion- Please limit the word count within 200 words.

Thank You
