# Supplementary material for: Assessment of healthcare financing by older adults during routine consultations in Cameroon
Source: PLOS Glob Public Health. 2026 Apr 24;6(4):e0006197. doi: 10.1371/journal.pgph.0006197 (PMC13108730; doi:10.1371/journal.pgph.0006197)
Supplement: Attachment — Submitted filename: Response to reviewers.docx [file pgph.0006197.s005.docx]

Dear Reviewers,

We sincerely thank you for the time and effort dedicated to reviewing our manuscript entitled “Assessment of healthcare financing by older adults during routine consultations in Cameroon”. We are grateful for the constructive comments and insightful suggestions that have greatly helped us improve the quality and clarity of our paper.

Below, we provide a detailed, point-by-point response to each of the reviewers’ comments. All modifications made in the revised version are visible in the “Revised Manuscript with Track Changes”. For references to page number and line numbers of the modifications, please refer to the clean version.

**Reviewer 1**

- **Comment 1:** Your study shows a 43.5% out of pocket expenditure. This is relatively very low compared to multiple other reports for Cameroon that put out of pocket expenditure above 70%. Unless a serious selection bias is to be considered, kindly state (or review) your definition of out-of-pocket health expenditure. Traditionally out of pocket expenditures accounts for payment outside of an insurance scheme. According to this, household spending including from family members which was not part of a health fund or insurance scheme of any kind is also out of pocket expenditure. In your paper, contributions for family were not considered as out of pocket

**Response:** We thank the reviewer for this insightful comment and the opportunity to clarify how out-of-pocket expenditure was calculated in our study.

In our analysis, we distinguished between two components of direct patient payments:

1. Direct individual expenditures (43.5%), which correspond to payments made by the patient, from his/her own resources, at the point of care for consultation, medication, transport, and other services.
2. Family or household contributions (47.1%), which represent the additional financial support provided by relatives or household members for the same episode of care.

This approach was designed to better understand the financial dynamics within households, rather than to exclude family contributions from the total health-related expenditure. Therefore, when considering both components together, the overall direct out-of-pocket expenditure, including family contributions—approaches national estimates (90.6%), which meet the Databank definition of out-of-pocket payments.

To address this concern, we have revised the Methods section (page 6, lines 133-135) to clearly describe these two categories and added a sentence in the results section (page 7 lines 170-172). We modified the abstract text accordingly (page 2 line 38).

- **Comment 2:** The high proportion of retired and inactive individuals contrasts with national data showing that most older adults remain economically active". Your statement above contrasts with your finding that 70.5% of participants had an income. Can you clarify this reference to a high proportion of retirees in your study population? Clear definitions of income categories will help.

**Response:** We thank the reviewer for this insightful comment, which helped us identify a point requiring clearer explanation.

It is important to note that retirees are not considered economically active in our paper, as their income derives from pensions or other retirement contributions rather than from active work. Accordingly, in our study, economic activity is limited to participants with income generated by active work, whether in the formal or informal sector (31.7%). which contrasts with national data.

To address this, we have reformulated in the Methods section the income categories used in our analytical framework: employment in the formal sector, employment in the informal sector, retired, and unemployed (page 7 lines 138 – 141).

The Results section (page 8, table 1) has been revised to explicitly describe these categories, and the Discussion has been updated to clarify this distinction and prevent further ambiguity (page 10, lines 203-206).

**Comment 3:** "The study further reveals signs of weakened family solidarity: only one-third of participants reported that all their children were financially independent, in a context marked by high youth unemployment. In such cases, the family traditionally considered a safety net—may in fact contribute to economic precarity".

Your statement above contrasts with your finding that health care spending for your participants was covered by families 47.1% (over 90% by children) only second to self-sponsored care. Can you please explain? In addition, family solidarity will not be defined by financial independence of the children. Unless you clarify and give context to this with comparative figures, this conclusion might be inappropriate.

**Response:** Thank you for this valuable comment. We agree that our formulation may have created some ambiguity. Our intention was not to equate family solidarity exclusively with the financial independence of adult children, but rather to illustrate how the traditional patterns of intergenerational support are being reshaped by economic pressures.

In our study, while family support remains significant in health care spending, this coexistence of support and dependency reflects a dual dynamic. On one hand, familial solidarity persists, particularly through children’s financial contributions to parents’ health care. On the other hand, this solidarity is strained by the economic vulnerability of younger generations, many of whom are unemployed or underemployed.

Thus, our statement on “weakened family solidarity” refers less to a decline in moral or emotional ties, and more to the erosion of the family’s capacity to function as a reliable safety net in a context of generalized economic precarity. We have clarified this distinction in the discussion and add comparative references to support this nuanced interpretation (pages 10-11, lines216 – 224).

**Reviewer 2**

**Comment 1:** The authors have conducted univariate and bivariate analyses, but no multivariate analysis. There is also a need for improving the language of writing to academic requirements. The authors are also suggested to use STROBE checklist for cross-sectional studies for adherence to reporting standards.

**Response:** We thank the reviewer for these constructive comments.

1. Regarding the statistical analysis: we acknowledge that including a multivariate analysis could further strengthen the robustness of our findings. However, given the exploratory design and the sample size limitations of our study, we opted for descriptive, univariate, and bivariate analyses to highlight the main patterns of healthcare financing among older adults. Our primary aim was to describe the distribution and relationships of key variables rather than to establish causal associations.
2. Regarding the language of the manuscript: the entire manuscript has been carefully revised for clarity, conciseness, and compliance with academic English standards. Grammatical and stylistic edits were made throughout to ensure readability and consistency with PLOS One guidelines. An English speaker reader revised the last version of the manuscript.
3. Regarding the STROBE checklist: we fully agree with the importance of adhering to reporting standards. We have also reviewed the structure of the manuscript to ensure alignment, when pertinent with STROBE recommendations for clarity and transparency in reporting. Modifications have been made to the Methods section for better understanding and reproducibility of the study. We have added the rationale for the recruitment sites (pages 4 - 5, lines 92 - 95), criteria for participation in the study (page 5-6, lines 101-107), sample size justification (page 5, lines 107-113).

**Reviewer 3**

- **Comment 1:** Introduction- address the need for the study. Any similar study in the past? Why was Yaounde chosen?

**Response:** No similar study was conducted in the Cameroonian context. Research about older adults and healthcare is mostly limited to the pathological profile and barriers to access to care. But not about the cost of healthcare expenses generated by an episode of illness, this is what makes the originality of our work (page 4 lines 74 – 75). The choice of the city of Yaounde was motivated by the fact that it is where the main study (Grand Age) is conducted, the availability of different levels of health care and its cosmopolitan character (page 4 lines 77 – 78).

- **Comment 2:** Table 1- Format properly. Remove the commas and insert decimal points for percentages; Table 2 – Also needs the same correction.; Table 1- Age column totals up to 99.9%.; Figure 1- Total percentage adds up to 131.3%.; Figure 2- Total percentage adds up to 109.8%.

**Response:** We have updated the manuscript in line with your comments.

- **Comment 3:** Discussion- Please mention the strengths and limitations in a paragraph exclusively. This should go at the end of the discussion, not at the beginning

**Response:** We thank the reviewer for this helpful suggestion. In the revised manuscript, we have added a dedicated paragraph at the end of the Discussion section presenting both the strengths and limitations of the study.

- **Comment 4:** Conclusion- Please limit the word count within 200 words.

**Response:** We appreciate the reviewer’s observation. The conclusion has been carefully revised to be more concise and focused.

We believe that the revised version of our manuscript now addresses all reviewers’ concerns and improves the overall scientific rigor and readability of the work. We appreciate the opportunity to revise our manuscript and remain at your disposal for any additional clarifications.

Thank you once again for your consideration.

Sincerely,

The authors
