# Supplementary material for: Assessment of healthcare financing by older adults during routine consultations in Cameroon
Source: PLOS Glob Public Health. 2026 Apr 24;6(4):e0006197. doi: 10.1371/journal.pgph.0006197 (PMC13108730; doi:10.1371/journal.pgph.0006197)
Supplement: Attachment — Submitted filename: Response to Reviewers RAC-160226(1).docx [file pgph.0006197.s006.docx]

Dear Reviewers,

We sincerely thank you for the time and effort dedicated to reviewing our manuscript entitled “Assessment of healthcare financing by older adults during routine consultations in Cameroon”. We are grateful for the constructive comments and insightful suggestions that have greatly helped us improve the quality and clarity of our paper.

Below, we provide a detailed, point-by-point response to each of the reviewers’ comments. No comments were made on the manuscript’s body, neither its content, nor its form; consequently, no changes were made. The response to the comment on Figures 1 and 2 can be found below.

**Reviewer 3**

**Comment 1:** Great work authors. All concerns have been addressed except the pathological profile (figures 1 & 2). Are those mutually exclusive events? The percentages are not totalling to 100.

**Response:** Thank you for your constructive comment, which will help readers better understand our work. To answer your comment, in Figure 1, the variable being sought was the reason for consultation, as expressed in the question: "What brings you for a consultation today?" The responses were then organized into systems for easy analysis. The events were therefore mutually exclusive in this case, resulting in a total percentage of 100. Typos introduced during the creation of Figure 1 have been corrected. In the case of the figure 2, the question addressed to participants was an open-ended question worded as follows: "Do you suffer from any other illnesses besides the one that brings you to consultation today?", to which several answers were possible. Some participants thus mentioned more than one condition, resulting in a total percentage in Figure 2 exceeding 100.

To address these concerns, Figure 1 (Fig1.tif) has been corrected and a legend has been added to the corpus under figure 2’ title to make the figure easier to understand. The changes can be found in the Results section and (page 8, 167).

We believe that this revised version of our manuscript addresses reviewers’ concerns and improves the overall scientific rigor and readability of the work. We appreciate the opportunity to clarify our work and remain at your disposal for any additional clarifications.

Thank you once again for your consideration.

Sincerely,

The authors
